# Numerous News Items

**Published:** 1894-03

**Authors:** 


### Medical News and Miscellany.

**Dr. S. S. Wallace** has moved from Clarendon, Texas, to Long Creek, Illinois.

---

**Our Say:**—The JOURNAL would like to correspond with any physician who wishes to buy an electrie battery or cabinet, new. Inducements offered.

---

**Married,** at Fort Worth, Texas, February 21st (ult.), Mr. Samuel T. Camp to Miss Tommie Adams, sister of Dr. W. A. Adams, all of Fort Worth.

---

**Those Who Are in Arrears** for subscriptions are earnestly requested to settle, at least in part. We are giving good value, and it takes oodles of money to do it. "Please remit."

---

**Professors James Kennedy and S. M. Morris,** of the Medical Department University of Texas, Galveston, have prepared and published a "Chart of the Official Chemical Salts, both inorganic and organic of the U. S. P., 1890; showing the methods of production, together with a schedule of Neutral Principles and Phenols," for the use of students. The JOURNAL acknowledges the receipt of a copy, and returns thanks.

**The Crime of Substitution.**—It is a courtesy to call substitution such outrageous, high-handed and wholesale swindling as that exposed in a letter from Lambert Pharmacal Co. published herewith. We are glad to see that there is a law that can reach these fellows; and we think Lambert Co. made a mistake in not prosecuting all of the parties caught in the act of selling a mixture of their own for Listerine. What a sorry lot the druggists of Chicago must be, really. Attention is called to the letter under Publishers' Department.

---

**Dr. J. M. Fry.**—The JOURNAL'S esteemed friend Dr. J. M. Fry, late of Elmo, Texas, writes us from away up in the natural gas regions of Indiana. He became dissatisfied with the part of Texas which had known him so long, and without consideration for his friends, pulled up and set out to find a better place. We are pleased to hear from the doctor that he has found a place to his liking, and hope that if he has not exactly "struck oil," he may, at least, strike gas, or pay dirt, or something that will pay; he deserves it. The doctor could not do without his copy of the red-back, and ordered it sent to Mays, Rush county, Indiana. Good luck to him.

---

**Myxœdema.**—Dr. J. P. Coffy, of Plano, Texas, an old time and ardent supporter of the TEXAS MEDICAL JOURNAL, reports to the JOURNAL a case of myxœdema in his practice, which he successfully treated with desiccated thyroid gland of the sheep. He gave fifteen grains each second day. After a few doses had been taken, the doctor says, there was perceptible change for the better, and within four weeks from beginning the treatment "the chilliness had entirely disappeared, the skin became moist, the bowels regular, and there was a reduction in weight of twenty-seven pounds. The mind was bright and the voice clear and strong."

Dr. Coffy makes the diagnosis the strong point in this case, and says, "The lady had consulted a number of physicians, myself included, and not one of us ever seemed to have had the remotest idea of the cause of all this trouble." In the report, the doctor, of course, detailed all the symptoms and gave a minute clinical history of the case (which we omit, as the reader can find the disease described in the later works). He frankly asks "was it ignorance or carelessness?" and as frankly acknowledges *both* on his part—at first. It was the first and only case, he says, he ever saw or ever heard of in Texas, and he had to "read up." The doctor expresses the belief that cases occur in Texas as elsewhere, but are not diagnosed,—and hence are treated for something else, and without success. We venture to assert that this is the first case successfully treated on the new plan by any of our Texas doctors.

---

Died, at Sabinal, Texas, February 25th, Mrs. Mary Ernest Cocke, wife of Dr. Lewis W. Cocke, of San Marcos. Mrs. Cocke was born and raised in Austin. Her maiden name was Mary Ernest, and her family are old residents, and highly respected in this city. Mrs. Cocke had been for some two years suffering with tubercular laryngitis, and developed pulmonary disease only comparatively recently. Dr. Cocke took her out to Sabinal cañon, in West Texas, about the first of February,—having heard reports of the wonderful climate and the still more wonderful mineral springs, in the hope of benefiting her health. After a stay of about ten days the doctor wrote us that his expectations were being realized, and that he had some hope of his wife's ultimate recovery; hence the news of her death, received here on the 26th, was a surprise.

Mrs. Cocke was a most estimable lady, much beloved by all who knew her, and her early death will be deeply deplored by a wide circle of attached friends. She leaves two young children, a son aged five, and a little daughter of only two and a half summers; both too young to realize their irreparable loss. The Journal extends heartfelt sympathy to the bereaved husband and to the family. Dr. Cocke will return to San Marcos and place his little ones in the care of his mother.

